# Benefits of early aneurysm surgery: Southern Iran experience

**Published:** 2012-11

**Authors:** Abdolkarim Rahmanian, Ehsan Ali Alibai, Mohammad Jamali, Ali Razmkon

**Affiliations:** ^a^Shiraz Neurosciences Research Center, Shiraz University of Medical Sciences, Shiraz, Iran.

## Abstract

**Background:**

Neurovascular surgery has been practiced in Shiraz, the main referral center of the Southern Iran, for over 30 years. However, because of the development of a subspecialization specialty of neurovascular surgery in Shiraz Department of Neurosurgery the trend has increasingly accelerated in recent years. The present study focuses on the description of techniques we are currently used for early clipping of ruptured intracranial aneurysms in the anterior circulation. Improvements in outcome, mortality and rebleeding rates are also addressed.

**Methods:**

Our previous and conventional strategy for management of aneurysmal subarachnoid hemorrhage (SAH) earlier than 2010 was delayed surgery using the pterional approach with an old neuroanesthetic protocol for most of patients. Since 2010, we have shifted to an early surgery program using the lateral supraorbital approach and advanced neuroanesthesia (the Helsinki protocol) for nearly all patients. The outcome data and preliminary results are presented here.

**Results:**

Mortality rate, which was about 40% in conventional approach, is now reported to be about 14%, regardless of the significant increase in the number of high grade patients. Furthermore, in the conventional method, acute phase of rebleeding occurred in 10.5% of patients, while in this new protocol the incidence is 1-2%, which occurs most frequently in patients with a delayed refer more than 72 hours for surgery.

**Conclusions:**

The establishment of the early-surgery approach for ruptured anterior circulation aneurysms through the lateral supraorbital approach along with specific anesthetic protocol has resulted in significant improvement of morbidity, mortality and rebleeding rates in our department.

**Keywords:**

Neurovascular surgery, Early-surgery approach, Aneurysmal subarachnoid hemorrhage


					Volume 4, Suppl. 1 Nov 2012
Publisher: Kermanshah University of Medical Sciences
URL: http://www.jivresearch.org
Copyright:
Congress of Iranian Neurosurgeons, 14-16 November 2012, Kermanshah, Iran
Paper No. 29
Benefits of early aneurysm surgery: Southern Iran experience
Abdolkarim Rahmanian a
, Ehsan Ali Alibai a
, Mohammad Jamali a
, Ali Razmkon a,*
a
Shiraz Neurosciences Research Center, Shiraz University of Medical Sciences, Shiraz, Iran.
Abstract:
Background: Neurovascular surgery has been practiced in Shiraz, the main referral center of the Southern Iran, for
over 30 years. However, because of the development of a subspecialization specialty of neurovascular surgery in
Shiraz Department of Neurosurgery the trend has increasingly accelerated in recent years. The present study focuses
on the description of techniques we are currently used for early clipping of ruptured intracranial aneurysms in the
anterior circulation. Improvements in outcome, mortality and rebleeding rates are also addressed.
Methods: Our previous and conventional strategy for management of aneurysmal subarachnoid hemorrhage (SAH)
earlier than 2010 was delayed surgery using the pterional approach with an old neuroanesthetic protocol for most
of patients. Since 2010, we have shifted to an early surgery program using the lateral supraorbital approach and
advanced neuroanesthesia (the Helsinki protocol) for nearly all patients. The outcome data and preliminary results
are presented here.
Results: Mortality rate, which was about 40% in conventional approach, is now reported to be about 14%,
regardless of the significant increase in the number of high grade patients. Furthermore, in the conventional method,
acute phase of rebleeding occurred in 10.5% of patients, while in this new protocol the incidence is 1-2%, which
occurs most frequently in patients with a delayed refer more than 72 hours for surgery.
Conclusions: The establishment of the early-surgery approach for ruptured anterior circulation aneurysms through
the lateral supraorbital approach along with specific anesthetic protocol has resulted in significant improvement of
morbidity, mortality and rebleeding rates in our department.
Keywords:
Neurovascular surgery, Early-surgery approach, Aneurysmal subarachnoid hemorrhage
* Corresponding Author at:
Ali Razmkon: Shiraz Neuroscience Research Center, Shiraz University of Medical Sciences, Shiraz, Iran, Tel: 09173148711,
Email: Ali.razmkon@gmail.com, (Razmkon A.).

				

